# DOE-SLAM: Dynamic Object Enhanced Visual SLAM

**DOI:** 10.3390/s21093091

**Published:** 2021-04-29

**Authors:** Xiao Hu, Jochen Lang

**Affiliations:** Faculty of Engineering, University of Ottawa, Ottawa, ON K1N 6N5, Canada; jlang@uottawa.ca

**Keywords:** vSLAM, AR, computer vision

## Abstract

In this paper, we formulate a novel strategy to adapt monocular-vision-based simultaneous localization and mapping (vSLAM) to dynamic environments. When enough background features can be captured, our system not only tracks the camera trajectory based on static background features but also estimates the foreground object motion from object features. In cases when a moving object obstructs too many background features for successful camera tracking from the background, our system can exploit the features from the object and the prediction of the object motion to estimate the camera pose. We use various synthetic and real-world test scenarios and the well-known TUM sequences to evaluate the capabilities of our system. The experiments show that we achieve higher pose estimation accuracy and robustness over state-of-the-art monocular vSLAM systems.

## 1. Introduction

Autonomous robots, augmented reality (AR), and indoor scene understanding systems [1] often rely on simultaneous localization and mapping (SLAM) [2] for navigation and scene reconstruction. They commonly operate in dynamic environments. The different sensors in SLAM systems [3], such as monocular cameras, stereo cameras, depth cameras [4], and IMU [5,6], influence how well the systems work in dynamic environments. Monocular visual SLAM systems (vSLAM) are particularly attractive because of their general availability and the simplicity of their cameras. Matching the scene over subsequent video frames, either directly or through features, allows vSLAM to succeed. If an object in the scene moves and hence the scene changes, the matching constraints become unreliable, and localization and mapping start to drift. Our focus in this study was on indoor monocular-based augmented reality systems where close dynamic objects often obstruct the camera’s view. This problem affects monocular vSLAM more than systems based on stereo cameras and depth sensors because, due to a scale ambiguity, the absolute depth of the scene remains unknown. The common strategy [7,8] to deal with dynamic objects in monocular vSLAM is masking all the moving objects to only track the background. Tracking the background alone provides better performance in some cases but also reduces the usable image content as some of the background is covered by dynamic objects. This can lead to localization and mapping failures.

Here, we present a new method for monocular vSLAM to fully exploit all features in each image frame including the features of dynamic objects. Monocular vSLAM is both inexpensive and monocular cameras are widely available. Some depth cameras rely on infrared (IR,) which is problematic in bright daylight environments. Other depth cameras rely on time-of-flight ranging, which can fail for uncooperative targets. Monocular cameras can operate in a wide range of conditions. We extend monocular ORB-SLAM2 [4] for adaptation to dynamic environments based on the assumptions that the moving objects in the scene are rigid bodies and that their motion is predictable over a few video frames. These assumptions are often satisfied as if an object is not rigid, we can often treat at least a part of the object as approximately rigid. Our system can successfully track the camera even in scenes where multiple humans obstruct the background for a short period as we demonstrate in our experiments. Based on these assumptions, our method provides the following contributions:

- We present a novel monocular vSLAM system with deep-learning-based semantic segmentation to reduce the impact of dynamic objects and use the dynamic features to improve the accuracy and robustness;
- We present a method to estimate camera pose and object motion simultaneously in addition to ORB-SLAM2 [4] for monocular cameras;
- We propose a strategy to recover the camera pose from the predicted object motion if a moving object obstructs enough background features, such that tracking from the background alone is impossible; and
- We generate a number of new test cases with ground truth for camera trajectory, object motion trajectories, and a semantic segmentation mask for each frame.

Our experimental setup relies on real-world test cases, the TUM RGB-D dataset [9], and various public 3D environments: the Replica-Dataset [10] and Matterport3D [11] in combination with self-made animations for evaluation. Our method has higher accuracy and robustness compared with state-of-the-art vSLAM systems.

## 2. Related Work

SLAM is widely used in AR [12] for registration as it is able to reconstruct a map of the surrounding scene and localize the device simultaneously without any prior knowledge of the environment. Although not all AR systems use vSLAM, monocular vSLAM is an important special case because of the simplicity and generality of only relying on a single camera. vSLAM is different from structure from motion (SfM). SfM, the same as SLAM, uses a collection of 2D images to reconstruct the 3D structure of a stationary scene [13]. SfM, however, focuses on reconstructing an exquisite 3D model of the scene [14], and the main goal of vSLAM is to navigate the device in the environment. Hence, vSLAM is usually required to operate in real-time, but SfM is not [15].

### 2.1. Classic SLAM

Classic SLAM can be divided into direct methods versus feature-based (indirect) methods [16]. Direct SLAM (e.g., LSD-SLAM [17] and DTAM [18]) uses all the information from the sensor without pre-processing. However, feature-based SLAM (e.g., ORB-SLAM [19] and PTAM [20]) pre-computes features before feeding them into the actual SLAM threads. The most common pre-computation for vSLAM is feature-points extraction in addition to semantic segmentation [21], optical flow regularization [22], and depth map prediction [23]. Deep learning is now commonly used in SLAM. Qiu et al. [24] used deep learning to detect loop closure. Tateno et al. [25] used a convolutional neural network (CNN) to predict depth during pre-processing and for semantic segmentation.

ORB-SLAM2 [4] works with three parallel threads of tracking, local mapping, and loop closure in order to accelerate the computation. It employs ORB descriptors in all threads, adopts the bag-of-words method to speed up feature matching, and uses bundle adjustment (BA) as the back-end optimization framework. A covisibility graph and an essential graph reduce the number of edges in the graph to accelerate the graph optimization step.

### 2.2. Dynamic SLAM

Saputra et al. [26] reviewed dynamic SLAM and categorized methods into background foreground initialization, geometric constraints, optical flow, ego-motion constraints, and deep learning. Many dynamic SLAM approaches rely on depth sensors. DynaSLAM [21], in its RGB-D mode, uses a low-cost tracking method to verify if each object is moving by calculating the depth change between two frames. The RGB-D dynamic SLAM system mid-fusion [7] uses mask R-CNN [27] to segment objects. Bârsan et al. [28] used a stereo camera as the input sensor and used the left image to segment objects, then calculated two-frame scene flow to verify object motion. Jaimez et al. [29] used an RGB-D input and k-means to cluster all points by depth, then estimated the scene flow to find moving objects. DetectFusion [30] also uses an RGB-D camera as input, where objects are segmented in the RGB image with the help of geometry segmentation in the depth image. They used residual masks from the iterative-closest-point (ICP) registration to detect object motion. EM-fuison [31] uses an RGB-D sensor as input, mask R-CNN to segment objects, and tracking and mapping of objects and the static background. It uses a probabilistic expectation maximization formulation to determine the unknown association of pixels with objects, and based on the association, to track multi-objects as well as camera pose for mapping. MaskFusion [32] is also an RGB-D SLAM system where mask R-CNN, in combination with a geometric segmentation algorithm, is used for instance segmentation. Co-Fusion [33] is an RGB-D SLAM system that stores each model as a set of 3D points. It uses a conditional random field (CRF) to segment motion, and tracks each object by minimizing a geometric error based on a dense ICP alignment with a photometric cost based on the stored 3D model in each frame. Fan et al. [34] presented the idea of fusing a sequence of frames that contain dynamic objects into a single frame image without dynamic objects. The stereo camera is used to delete dynamic regions at each time frame. All dynamic regions are deleted in each frame before fusing them into the overall static image frame. DDL-SLAM [35] is an RGB-D SLAM system for dynamic environments. DDL-SLAM adopts deformable U-Net [36] to provide pixel-wise semantic segmentation followed by multi-view geometry to verify if an object is dynamic. All the dynamic features are discarded, and only the background features are used for camera localization. All these dynamic SLAM approaches are not directly applicable to monocular vSLAM, which was our focus.

Some researchers investigated back-end optimization. Sünderhauf et al. [37] employed switchable constraints to deal with false-positive loop closure constraints, which increased the robustness of the overall SLAM system. Olson et al. [38] proposed characterizing the error of those loop closures in a fully integrated Bayesian framework to account for their non-Gaussian behavior. Agarwal et al. [39] used a robust function that generalizes classical gating and dynamically rejects outliers, which speeds up the optimization process. Lee et al. [40] considered low-dynamic environments using a pose graph structure to prune false pose graph constrains.

DS-SLAM [41] adopts SegNet [42] to separate foreground objects from the background. They use the epipolar line constraint to verify if an object is moving. All the moving features are ignored in the tracking step. Tan et al. [43] detected changes that occur in the scene by projecting the map features into the current frame for appearance and structure validation. DynaSLAM for monocular vSLAM relies on mask R-CNN to segment objects in video frames. When using a monocular camera, DynaSLAM ignores all features on the segmented objects and only uses background features for tracking. We compared our method with DynaSLAM, finding an improvement in accuracy and robustness. Dynamic-SLAM [44] employs a similar strategy as DynaSLAM to use a deep-learning-based method to detect a dynamic object, and then only the background features are used for tracking and localization. Dynamic-SLAM is also compared with our system by using the TUM dataset in Section 4.3.

## 3. Method

**Figure 1:**
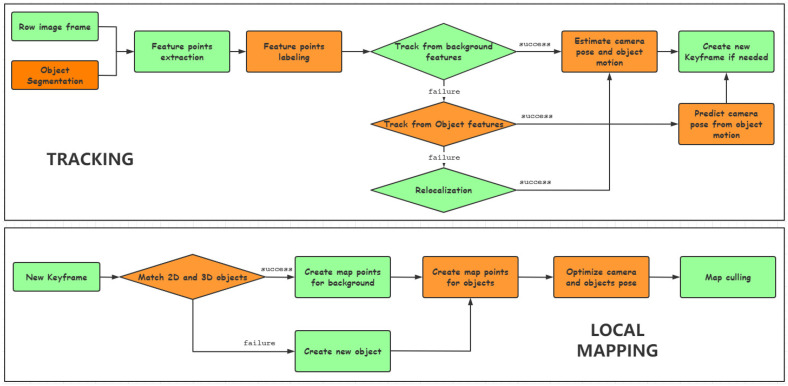
Tracking (**top**) and local mapping (**bottom**) of DOE-SLAM. The changes compared with ORB-SLAM2 are shown in orange boxes.

We built our DOE-SLAM system relying on a monocular camera as the only sensor motivated by the cameras being inexpensive and readily available. We performed modifications mainly of tracking and local mapping for which the flow diagram of DOE-SLAM is shown in Figure 1. The flow diagram shows the case of at least one detected foreground object in the current frame. For static scenes, DOE-SLAM works exactly the same as ORB-SLAM2.

**Figure 2:**
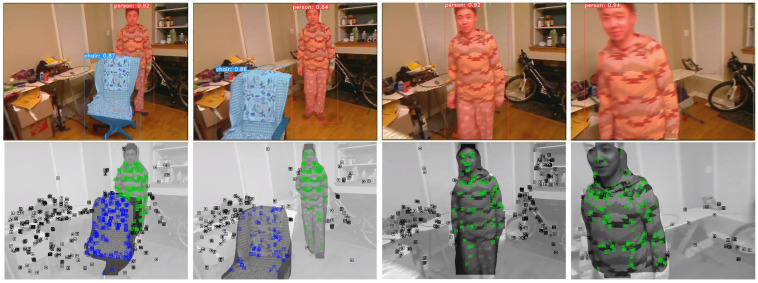
A scene with multiple moving objects. The first row shows the original frames with the semantic masks from YOLACT [45], and the second row shows the tracking result from DOE-SLAM. There are two moving objects: an armchair and a person.

**Figure 3:**
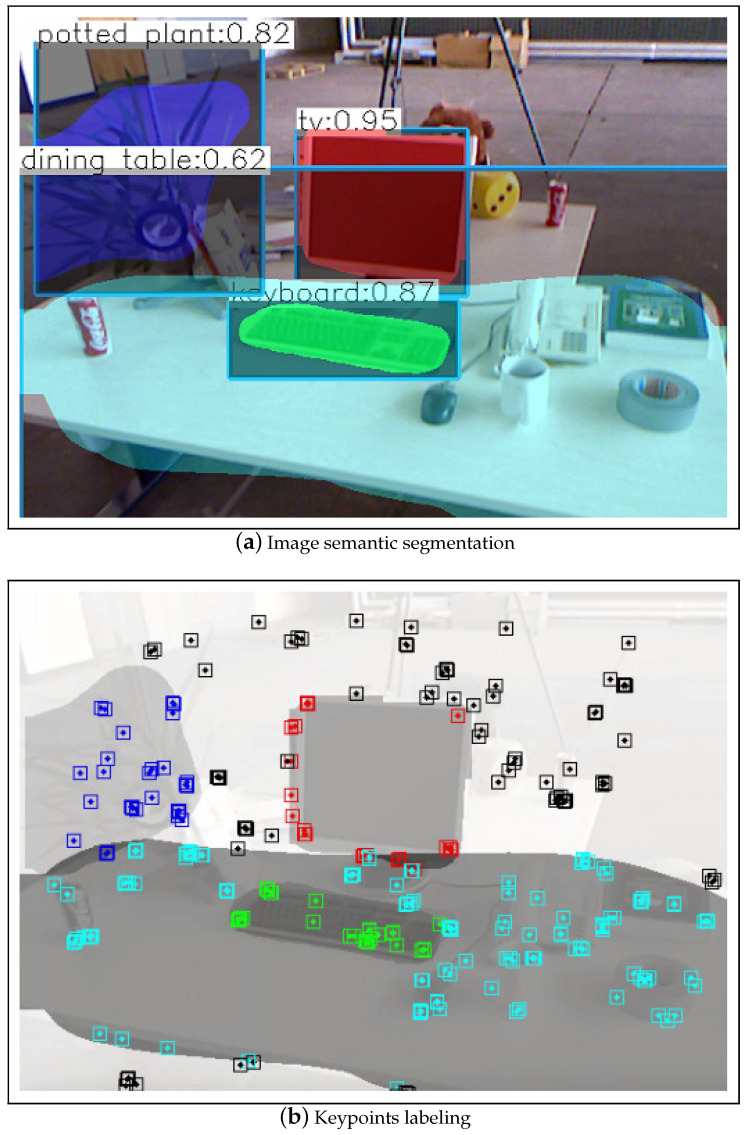
Object modeling (image from TUM dataset). The result of semantic segmentation masks from mask R-CNN [27] (**a**), and labeling the ORB keypoints based on the semantic information (**b**).

**Figure 4:**
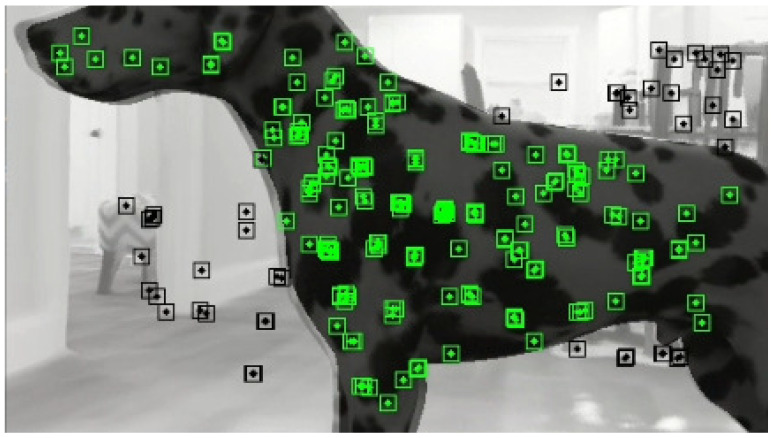
A moving object (Dalmatian dog) covers most of the frame.

Tracking is the main contribution of DOE-SLAM. We tested YOLACT [45] (Figure 2), mask R-CNN [27] (Figure 3), and manually labeling (Figure 4) as well to provide segment information for each frame. The input to DOE-SLAM is a sequence of images from a mono camera and their corresponding segmented object masks. In the feature extraction step, our method provides each feature point a class ID, marking if it belongs to a foreground object or the background. Then, the background features are used to estimate the camera pose. If tracking succeeds, the object pose is estimated afterward. Otherwise, the camera pose is predicted from the object pose.

Additional steps are added into the local mapping thread to deal with object point cloud modeling and object motion estimation. First, matches are found between objects in the keyframe (2D features) and objects in the map (3D map points). If matches are found, new map points are created and added for the matched objects. Otherwise, the system creates a new object in the map. Once the map points are created, the camera and object pose are optimized separately if the object is moving. Our DOE-SLAM uses slightly more memory compared with ORB-SLAM2. The memory added contains a position matrix for each object and a class label for each map point. In ORB-SLAM2, the world map class stores all the references of the map points. In our DOE-SLAM, the world map class stores all the references of the objects, and each object stores the references of all the map points that belong to this object. The number of the map points remains unchanged; hence, the memory requirements of our method and ORB-SLAM2 are similar.

### 3.1. Object Modeling

As an object may move but the background is always static, it is necessary to separate object features from background features. Given an image frame and its corresponding segmentation masks, we first extract all ORB feature points. Then, based on the semantic segmentation, each feature point is labeled with a class index marking to which object it belongs. The background is treated as an object. The system first tries to estimate the camera pose only based on background features. If the estimation succeeds, our system attempts to find the set of matched object feature points pair Γ in two frames based on ORB feature matching. Let $o_{i}^{a}$ denote the *a*th object in frame *i* and $p_{i}^{x}$ represent the *x*th feature point in the frame *i*. Define the function c(p) to return the object to which feature point *p* belongs; $c\left(p_{i}^{x}\right)=o_{i}^{a}$ if $p_{i}^{x}$ belongs to $o_{i}^{a}$. Given frame $f_{i}$, the subsequent frame $f_{j}$ and $\gamma =(p_{i}^{x},p_{j}^{y})$ representing the element in Γ, we have the following method to match the object:

(1) $$\begin{array}{c} BId\left(o_{i}^{a},f_{j}\right)=\underset{b}{argmax}\sum_{\gamma \in \Gamma }match\left(\gamma ,a,b\right) \end{array}$$

$$match\left(\gamma ,a,b\right)=\left\{\begin{array}{cc} 1,\; & if\;c\left(p_{i}^{x}\right)=o_{i}^{a}\wedge c\left(p_{j}^{y}\right)=o_{j}^{b}|\gamma =\left(p_{i}^{x},p_{j}^{y}\right)\\ 0,\; & otherwise \end{array}\right.$$

For each object $o_{i}^{a}$, our system finds the best matching object $o_{j}^{b}$ in $f_{j}$ by calculating $BId(o_{i}^{a},f_{j})$. If BId>τ, we consider that $o_{i}^{a}$ matches $o_{j}^{b}$. We define the threshold τ as three-quarters of the number of feature points belonging to $o_{j}^{b}$ in $f_{j}$.

Once an object is matched, the system estimates the object pose, builds new object map points, and calculates the object motion in the following period. Figure 3 shows the result of our object modeling method. We tested the method on the TUM RGB-D dataset [9] with mask R-CNN [27] to segment the image. There were five objects in the image: monitor (red), keyboard (green), plant (blue), desk (cyan-blue), and the background (black). To avoid confusion from masks overlapping, we first sorted the object masks by size; then, for each feature point, we found the smallest mask to which it belonged.

### 3.2. Object Motion Estimation and Optimization

Our system tracks the camera pose and the object motion simultaneously. In the SLAM problem, the main target is to estimate the transformation matrix between the world and the camera coordinate systems. However, in dynamic environments, the motion uncertainty of the feature points influences the estimation. There may be multiple moving objects in the frame, and DOE-SLAM handles tracking multi-objects in the scene. Object motion estimation interconnects with camera pose estimation. We need to know $T_{w}^{c}$ before calculating the object motion in the world frame $T_{w}^{o}$. We write $T_{w}^{c}$ to express the transformation that transforms a point from world to camera coordinates, $T_{w}^{o}$ to present the transformation from world to object coordinates, and $T_{c}^{o}$ to represent the transformation from camera to object coordinates. Thus, we first estimate $T_{w}^{c}$ from background features. Then, we measure $T_{c}^{o}$ only relying on object features and calculate the object–world transformation matrix $T_{w}^{o}=T_{c}^{o}\;T_{w}^{c}$. The motion of the object from frame $f_{i}$ to $f_{j}$ can be calculated as ${}^{i\to j}T_{w}^{o}$ = ${}^{j}T_{w}^{o}$${{(}^{i}T_{w}^{o})}^{-1}$. As we define ${}^{0}T_{w}^{o}=I$ (the identity matrix), if the object is static from the beginning to $f_{i}$, we have the equation ${{(}^{i}T_{c}^{o})}^{-1}$ = ${}^{i}T_{w}^{c}$. Similarly, if the object is static from $f_{i}$ to $f_{j}$, the motion of the object satisfies ${}^{i\to j}T_{w}^{o}=I$. So, we can use the difference between ${}^{i\to j}T_{w}^{o}$ and *I* to verify if the object moves following Equation (5).

Optimization is an important step in minimizing the error due to scale drift between each frame. If we optimize $T_{o}^{c}$ and $T_{w}^{c}$ separately from the beginning, the scale difference between background and objects increases. To lower the scale difference, we separate the optimization only if the object moves in the local map. Our system uses hysteresis thresholding to detect whether the object is moving; $\tau_{start}$ is used to detect when the object starts moving, and $\tau_{maintain}$ to test if the object keeps moving. $mScore_{t}$ for translation and $mScore_{r}$ for rotation are estimated and compared with $\tau_{tran}$, $\tau_{rot}$ separately. We consider the corresponding translation submatrix *t* and rotation submatrix *r* of *T*.

(2) $$\begin{array}{cc} \; & mScore_{t}{=\|}^{i\to j}t_{w}^{o}{\|}_{2};\;\;mScore_{r}={{\|}^{i\to j}r_{w}^{o}-I\|}_{2} \end{array}$$

(3) $$isRotate{(}^{i\to j}r_{w}^{o})=\left\{\begin{array}{cc} True,\; & if\;isMoving{(}^{i}T_{w}^{o})\;\\ \; & and\;mScore_{r}>\tau_{rot\_main}\\ True,\; & if\;not(isMoving{(}^{i}T_{w}^{o}))\;\\ \; & and\;mScore_{r}>\tau_{rot\_start}\\ False,\; & otherwise \end{array}\right.$$

(4) $$isTrans{(}^{i\to j}t_{w}^{o})=\left\{\begin{array}{cc} True,\; & if\;isMoving{(}^{i}T_{w}^{o})\;\\ \; & and\;mScore_{t}>\tau_{tran\_main}\\ True,\; & if\;not(isMoving{(}^{i}T_{w}^{o}))\;\\ \; & and\;mScore_{t}>\tau_{tran\_start}\\ False,\; & otherwise \end{array}\right.$$

(5) $$isMoving{(}^{j}T_{w}^{o})=isTrans{(}^{i\to j}t_{w}^{o})\;or\;isRotate{(}^{i\to j}r_{w}^{o})$$

Based on the above methods, DOE-SLAM is also able to handle scenes with multiple moving objects. Figure 2 shows a scenario where DOE-SLAM works in a scene that contains multiple moving objects. The first row shows the original frames from the video, and the second row displays the tracking state of DOE-SLAM. Two moving objects are in the first two frames, an armchair and a person. DOE-SLAM simultaneously tracks and creates models of two moving objects. In the third frame, the armchair has moved out of the scene. The person is tracked successfully. In the last frame, most of the key points are located on the person with a few of them on the background. DOE-SLAM can use the pose of the moving object to recover the camera pose as introduced in Section 3.3, whereas ORB-SLAM2 becomes lost.

### 3.3. Camera Pose from Object Motion

In the scenario shown in Figure 4, the camera cannot capture enough background features, which prevents successful background–camera pose estimation in the current frame, or even worse, the mapping. However, the features on the object are plentiful for object–camera pose $T_{c}^{o}$ estimation. To solve this problem, we designed a camera-pose prediction method to predict the camera pose from the object motion. As there may be several moving objects in the scene, DOE-SLAM can recover the camera pose from any tracked object. The first step is predicting the current object motion ${{(}^{i\to j}T_{w}^{o})}_{pred}$ from the object motion model recorded in previous frames.

We assume that the acceleration is small between two frames and use a constant velocity motion model to predict the object motion. According to this, our system estimates the camera pose in $f_{j}$ from $f_{i}$ as follows:

(6) $$\begin{array}{cc} {}^{j}T_{w}^{c} & ={{(}^{i\to j}T_{w}^{c})}_{pred}{\;}^{i}T_{w}^{c}\\ \; & {=}^{i\to j}T_{o}^{c}\;{{(}^{i\to j}T_{w}^{o})}_{pred}{\;}^{j}T_{w}^{c} \end{array}$$

As the model of the moving object is built separately from the static scene, the object may use a different scale factor than the background. In monocular vSLAM, the real scale of the scene is unknown. As a monocular camera cannot capture the real depth of the scene, the scale factor is set by the system during the initialization step. Define the scale factor difference between the background and the foreground moving object is $\Theta =S_{obj}\;S_{bkg}^{-1}$, where $S_{obj}$ represents the scale matrix of the object and $S_{bkg}$ represents the scale matrix of background. The object motion ${}^{i\to j}T_{w}^{o}$ estimated in Section 3.2 is actually a motion on a different scale than the background; hence, to express the object motion at the same scale with the background, we need to use the scale difference $\Theta^{-1}{\;}^{i\to j}T_{w}^{o}\;\Theta$. Considering the scale difference Θ, we can rewrite the previous prediction Equation (6) as follows:

(7) $$\begin{array}{cc} {}^{j}T_{w}^{c} & =\left(\Theta^{-1}{\;}^{i\to j}T_{o}^{c}\;\Theta \right)\;\left(\Theta^{-1}\;{{(}^{i\to j}T_{w}^{o})}_{pred}\;\Theta \right){\;}^{i}T_{w}^{c}\\ \; & =\Theta^{-1}\;{{(}^{i\to j}T_{w}^{c})}_{pred}\;\Theta {\;}^{i}T_{w}^{c} \end{array}$$

The final equation shows that there is a similarity transformation between objects and the background, which influences the prediction. Once the object moves out of the screen and enough background features can be captured, the system closes the scale difference by local BA. Only the frames fully covered by the moving object suffer this scale variance, but a scale difference is preferable to losing tracking.

The potential error in our prediction method accumulates over time; however, in most scenarios, it is reasonable that the method improves localization. If the object moves fast, there are only a few frames with not enough background features, so we have to rely on object motion. However, if the object moves slowly, the object does not influence the estimation too much as the object is nearly static. The system can build an object model that shares the same scale with the background, which means Θ≈I.

## 4. Experiments

We compared DOE-SLAM with state-of-the-art approaches in terms of accuracy and robustness. We generated our own test datasets as DOE-SLAM is designed for dynamic environments, so we needed to evaluate the estimation of not only the camera pose but also the object motion. We selected the Replica-Dataset [10] and Matterport3D [11] to generate the background environment. We used Unity3D to produce the test datasets with dynamic objects in those environments. We also report the results of using our method on two real-world test scenarios.

We contrast DOE-SLAM with ORB-SLAM2 as it forms the basis of our approach and with DynaSLAM to demonstrate the effectiveness and accuracy of our approach. Some TUM datasets [9] were also selected to better illustrate the performance of our system. We evaluated the accuracy in terms of root mean square error (RMSE) [46] of the absolute trajectory error (ATE) and the robustness by counting the number of frames when tracking was lost. Our experiments were divided into four parts; in Section 4.1, we consider the situation where the object in the view is static at first and then starts to move; in Section 4.2, we consider when the object passes through the view without stopping; in Section 4.3, several selected TUM datasets with human motions are employed to represent common scenarios; and in Section 4.4, two real-world scenarios are tested. All algorithms were tested on an Intel(R) Core(TM) i7-8700 CPU@ 3.20 GHz, GTX 1070 GPU with 16 GB RAM running Ubuntu 16.04.

### 4.1. Motion of Previously Static Objects

We performed tests with a previously static object starting to move while in the view of the camera. We speculate that this situation commonly occurs, e.g., if a person or pet rests then gets up and moves as the camera comes closer. We tested three different scenarios: in the first scenario, ORB-SLAM2 does not lose tracking but accuracy may be affected; in the second scenario, ORB-SLAM2 may lose tracking but can typically re-localize quickly based on previous views; in the last scenario, ORB-SLAM2 typically requires re-localization and loop closure.

**Figure 5:**
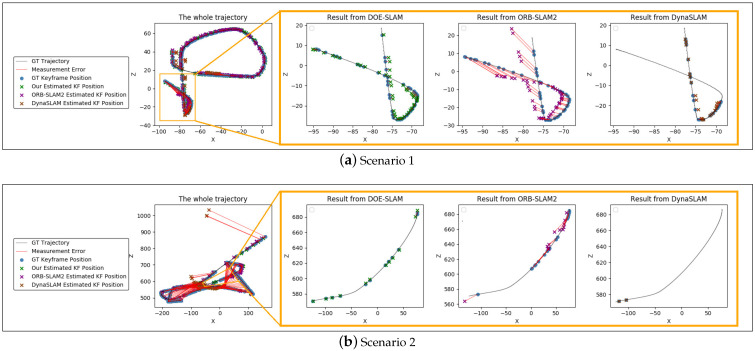

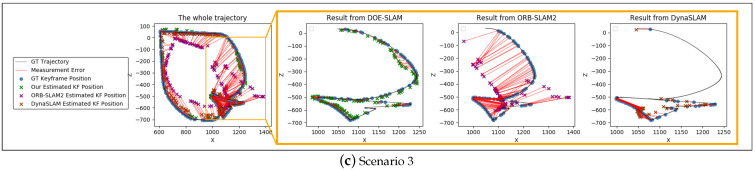
Motion of previously static objects. Overview of complete trajectories with marked successful tracking steps for DOE-SLAM, ORB-SLAM2, and DynaSLAM, and zoom-in to the region where the moving object is in view.

In Scenario 1, a foreground object covered most of the view and hence features on the object are tracked. Our test case sequence contained 1974 frames. The test results of DOE-SLAM, DynaSLAM, and ORB-SLAM2 for this scenario are shown in Figure 5a and Table 1a. The object was static at the beginning and the estimations from ORB-SLAM2 and DOE-SLAM were almost the same. DynaSLAM lost tracking quickly due to the lack of background feature points. However, when the object started to move, shown in the close up in the zoom-in graphs in Figure 5a, the estimated trajectory by ORB-SLAM2 drifted while DOE-SLAM was able to keep track of the position of the camera and was nearly unaffected. We observed that if a moving object dominated the view, ORB-SLAM2 may not get lost but the camera location was not reliable. In ORB-SLAM2, the features located on the background are treated as outliers because there are fewer of them than the features on the foreground object. In this case, ORB-SLAM2 keep stracking but the accuracy is affected. We also calculated the RMSE of the object pose estimation (Table 1a). The accuracy of the object pose estimation was lower than of the camera pose estimation. In many of the frames, the object contained fewer features than the background or the camera was unable to see enough background; hence, we estimated the object motion with some error.

In Scenario 2, a foreground object again covers most of the view; hence, features on the object will be tracked. However, this time, the occlusion is severe enough so that without dynamic object handling, camera pose estimation will fail. We generated the test case from the Replica-Dataset in Unity to assess this scenario with 1596 frames. Figure 5b and Table 1b show the results obtained for this scenario. The object is moving during the trajectory in the orange box. The result from ORB-SLAM2 shows that when the object started to move, the camera pose estimation started to be affected. After some frames, tracking in ORB-SLAM2 failed completely, as can be seen from the part of trajectory curve without estimated blue camera positions. After the moving object passed by, the system re-localized immediately as the camera had visited this location before. Conversely, our DOE-SLAM not only always continud tracking, but also estimated the trajectory with good accuracy. Table 1b provides the RMSE and the number of lost frames for ORB-SLAM2, DynaSLAM, and our DOE-SLAM. In this scenario, our method outperformed both methods in terms of accuracy and robustness.

Scenario 3 contained a longer period where the moving object blocked the camera view of the background. The scenario also provided the opportunity for loop closure after the moving object moves out of the view. The test case was generated from the Matterport3D dataset with 2385 frames. The results are shown in Figure 5c and Table 1c. We ran the scenario multiple times; sometimes, ORB-SLAM2 would track from the object without getting lost but the accuracy was quite poor. However, most of the time, ORB-SLAM2 directly stopped tracking until a loop closure occurred. Similarly, DynaSLAM became lost in the area with the moving object marked by the orange box. In contrast, our DOE-SLAM worked well in this scenario. Despite the scale drift in frames when the object was in view, loop closure eventually corrected and minimized this error. Table 1c shows that our DOE-SLAM performs significantly better than ORB-SLAM2. The table also shows that the RMSE for ORB-SLAM2 was lower when tracking was lost than if the method continued tracking. DynaSLAM had a low RMSE due to calculating the RMSE in matched time steps only, but if the system is lost when significant error occurs, this leads to a reduced overall error. In all of these three test cases, the RMSE of DynaSLAM was lower than of ORB-SLAM2, and close to our DOE-SLAM. However, DynaSLAM was far less stable than ours in these test cases. When the foreground moving object obstructed the camera, tracking was easily lost. Our system performed better than both DynaSLAM and ORB-SLAM2 as it continued tracking successfully in these scenarios. We also compared the computational time required by DOE-SLAM with that of ORB-SLAM2. Table 2 shows the average operation time for each frame. The time usage was counted excluding the semantic segmentation operation.

### 4.2. Fully Dynamic Object

We also created scenarios where a moving object suddenly appeared in view but the object was always in motion when seen by the camera. As the moving object becomes dominant in the view, the scale estimation for a mono camera is impacted, which may lead to scale drift between the map before the dynamic object comes into view and when the object leaves the view. Loop closure can eliminate scale drift; hence, we generated two test cases, one without and one with a loop.

**Figure 6:**
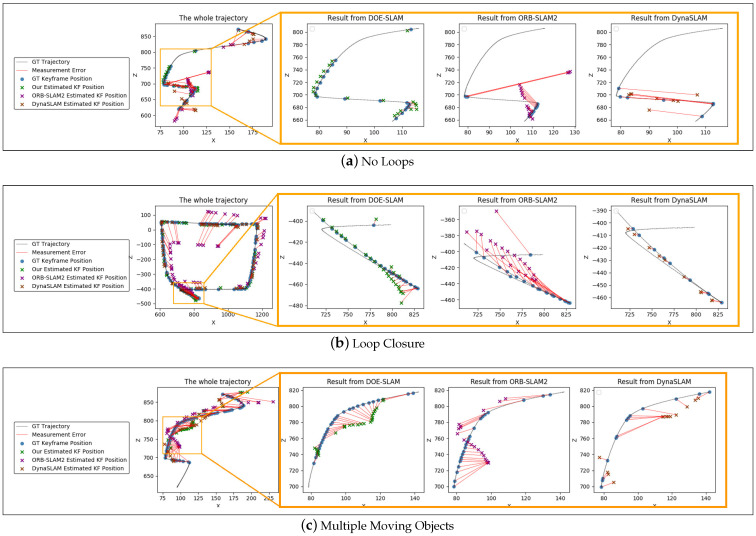
Fully dynamic scenes. Overview of complete trajectories with marked successful tracking steps for DOE-SLAM, ORB-SLAM2, and DynaSLAM and a magnification of the region where the moving object is in view.

We created the test case without a loop from the Replica-Dataset with 614 frames (Figure 6a). The graph shows that ORB-SLAM2wasis heavily influenced by the moving object. DynaSLAM not only became lost for some frames but also only tracked a few features before and after the lost tracking period. Our DOE-SLAM produced a high-accuracy trajectory. We generated another test case but this time with a loop from Matterport3D with 976 frames in total. Figure 6b shows that ORB-SLAM2 produced large errors over the complete trajectory. Although the results from DynaSLAM were accurate, DynaSLAM lost tracking during the period when the object obstructed the camera.

In order to test the performance with multiple moving objects, Scenario 6 was generated with the same scene as Scenario 4 but one more moving object was added. Figure 6c shows the result of Scenario 6 for the different systems. The moving object usually reduced the number of static background features. Compared with Scenario 4, adding one more moving object did not affect ORB-SLAM2 since there were still many features in the scene. DynaSLAM performed worse with higher error and more frames lost due to the reduced number of background features. The accuracy of DOE-SLAM was also affected because the number of static features determines the quality of the motion estimation of moving objects. However, DOE-SLAM still performed the best compared with the other two methods, as shown in Table 3.

Each test case on each system was tested five times and the average RMSE of the ATE (Table 3) shows that our DOE-SLAM outperformed the two comparators, having lower error and fewer frame lost. Scenario 4 contained no loop in the trajectory, Scenario 5 contained a loop, and Scenario 6 contained multiple moving objects. We also observed the average number of frames where tracking was lost. In these two test cases, our DOE-SLAM had the highest accuracy with the lowest RMSE and high robustness, as it did not lose tracking in any frame. Although ORB-SLAM2 did not get lost, it treats the object as always static and uses object features for tracking, which seriously affects the accuracy. Conversely, DynaSLAM outperformed ORB-SLAM2 on accuracy but it was unstable because it excludes object features from the camera pose estimation.

### 4.3. TUM Dataset

To convincingly evaluate DOE-SLAM in common scenarios, some TUM datasets were selected as test cases. As our system aims to deal with the dynamic object problem, we only picked the datasets that contain dynamic objects in the TUM dataset to show the improvement produced by our system. The results from Dynamic-SLAM are also listed as provided in the original paper, and compared in this section.

Table 4 lists the test results on selected TUM datasets. For each test case, we tested five times on each system and calculated the average. From the table, we can see that our DOE-SLAM outperforms achieved the lowest RMSE of ATE in the test cases (walking_halfsphere, sitting_rpy, and walking_xyz). In sitting_xyz, Dynamic-SLAM obtained the lowest RMSE of ATE, and our DOE-SLAM achieved a comparable result. However, in some cases, DynaSLAM performs better. In the walking_static test case, the camera is static, and there are many background features that can be captured. DynaSLAM obtained the lowest average RMSE in this test case as DynaSLAM dilates masks to cover more features near the edge area. Although humans are not rigid, we may still treat the human body as a mainly rigid body for a short period time for the localization in our system as demonstrated by these results. Table 5 shows the average execution time for processing one frame in selected TUM datasets in ORB-SLAM2, DynaSLAM, and DOE-SLAM. ORB-SLAM2 was the fastest since it ignores the effect of dynamic objects. DOE-SLAM and DynaSLAM achieved comparable results. Similar to DynaSLAM, DOE-SLAM tracks the motion of dynamic objects to improve robustness and accuracy.

In some test cases, the moving object is not the only issue that weakens the estimation. In walking_xyz, walking_halfsphere, and sitting_xyz, the camera pose measurement also suffered from the lack of features due to textureless surfaces because the camera only captures the white wall or the floor in several frames. DynaSLAM performed even worse than ORB-SLAM2 in these scenarios. It masked out the object feature points, and the background scene was textureless, which led to an insufficient number of features for tracking. In walking_rpy and sitting_rpy, the camera rotated along the principal axes (roll-pitch-yaw) at the same position [9]. Pure rotation is another issue that limited the accuracy of estimation in this scenario in addition to moving objects. The result for these two test cases showed that DOE-SLAM performed the same as DynaSLAM or worse. As we predict the camera pose from object motion, DOE-SLAM continued tracking in the challenging period, whereas DynaSLAM became lost. So, DOE-SLAM has a higher overall ATE.

The number of tracked frames is provided in Table 6. Moving objects affect the initialization of ORB-SLAM2, as the moving features confuse the image alignment. For DynaSLAM and DOE-SLAM, masking out moving objects reduces the usable feature points for initialization. In the TUM datasets, three SLAM systems are initialized and start tracking from different timestamps. Thus, we counted the total number of frames the system tracked instead of the number of lost frames. The table shows that in walking_halfsphere and sitting_xyz, all three systems continued tracking during the whole dataset. For walking_static, walking_rpy, and sitting_rpy, DOE-SLAM performed same as ORB-SLAM2 and tracked more frames than DynaSLAM because DynaSLAM masked out too many feature points, which produced unstable tracking. As for the walking_xyz test case, there are many feature points in the background, so DynaSLAM preformed similarly to DOE-SLAM and better than ORB-SLAM2, even though it ignores the moving feature points.

### 4.4. Real-World Test Cases

In order to fully explore the usability of our DOE-SLAM, we also recorded two videos of a real-world scene for testing. Due to the limitation of our devices, the ground truth in the form of the absolute trajectory was not captured. In this section, the relative results from different SLAM systems are compared. For each tested SLAM system, the estimated frame position was stored, and a rough trajectory was generated by smoothing the line chart of the estimated frame position set.

**Figure 7:**
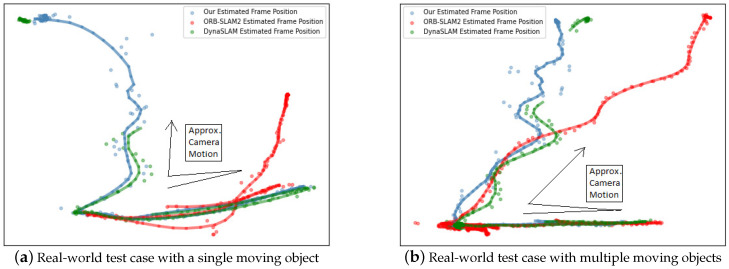
The estimated position of frames in real-world test cases by ORB-SLAM2, DynaSLAM, and DOE-SLAM. In both scenarios, the exact ground truth trajectory was unknown, but the general motion was from left to right and back to the left, followed by a forward motion.

Figure 7 shows the result from different systems. The black arrow in the graph shows the approximate trajectory of the camera motion. In both two test cases, the camera moved horizontally in the beginning to ensure that all the SLAM systems could initialize successfully. In this period, all three systems worked well, and the estimated trajectories were similar. The camera then moved forward, producing a vertical trajectory in the graph. Due to the effect of moving objects, trajectories from different systems started to diverge. There was a gap in the trajectory estimated by DynaSLAM (the green trajectory), since it lost tracking during the test cases. The trajectory estimated by DOE-SLAM (the blue trajectory) was closer to the trajectory estimated by DynaSLAM than to the trajectory estimated by ORB-SLAM2 (the red trajectory). Since DynaSLAM only tracks from static background features, the estimation was more accurate than that of ORB-SLAM when the tracking was not lost. DOE-SLAM and DynaSLAM both reduced the effect from moving objects. When DynaSLAM relocalized the camera at the very end, the position estimated was close to that of our DOE-SLAM. The trajectory estimated by ORB-SLAM2 drifted significantly due to ORB-SLAM2 tracking from the dynamic features without accounting for the modeling motion. The number of frames where tracking was lost was also counted, as shown in Table 7. In conclusion, our DOE-SLAM is more robust and accurate in dynamic scenes.

## 5. Conclusions

We constructed DOE-SLAM, a monocular vSLAM system that builds on ORB-SLAM2. DOE-SLAM is designed to work in dynamic environments where moving objects may obstruct the view of the static background (e.g., in indoor augmented reality systems). Once tracking of the background is lost, DOE-SLAM can automatically predict the camera pose from a tracked moving object with the motion estimated based on previous frames. Segmented image masks are used to separate moving objects from the background. Unlike competing methods, we use the object to localize the camera, which made our system more accurate and robust than the state-of-the-art DynaSLAM and ORB-SLAM2 in our test scenarios. Our results on selected public TUM datasets and our own test scenarios showed that our improvements translate to realistic real-world scenarios.

Two limitations exist in the present work. First, the dynamic object has to be detected; otherwise, our system cannot estimate its motion, and hence, the camera pose cannot be predicted from it. Second, the accuracy of our system decreases with the duration of the obstruction of the camera by the dynamic object, because the scale ambiguity of the monocular camera and the uncertainty of the object motion affect the estimation. To overcome these limitations, a direction for future work is to investigate the use of state-of-the-art object motion detection methods, then embed them into our system, and estimate a more detailed motion model.

Our work is expected to be beneficial in practice. We think that our work can be used in indoor AR navigation systems, as the camera is often obstructed by dynamic objects like pedestrians. AR-supported manufacturing, maintenance, and repair are other potential fields in which our work can be applied. In these tasks, moving components and the human body often obstruct the camera during operation. 

## Figures and Tables

**Table 1:** Comparison of the RMSE of ATE (cm) for the camera and object, with the number of lost frames for three scenarios.

**(a) Scenario 1**			
	**RMSE**	**Object RMSE**	**Lost Frames**
ORB-SLAM2	4.62	n/a	**0**
DynaSLAM	2.17	n/a	922 (46.7%)
DOE-SLAM	**1.67**	5.91	**0**
**(b) Scenario 2**			
	**RMSE**	**Object RMSE**	**Lost Frames**
ORB-SLAM2	23.34	n/a	39 (2.44%)
DynaSLAM	86.19	n/a	197 (12.34%)
DOE-SLAM	**18.05**	30.88	**0**
**(c) Scenario 3**			
	**RMSE**	**Object RMSE**	**Lost Frames**
ORB-SLAM2	176.46	n/a	**0**
ORB-SLAM2 (lost)	60.97	n/a	554 (23.23%)
DynaSLAM	**39.44**	n/a	639 (26.79%)
DOE-SLAM	48.66	53.40	**0**

**Table 2:** Comparison of the average computation time (ms).

	Scenario 1	Scenario 2	Scenario 3
ORB-SLAM2	20.5	25.06	20.65
DynaSLAM	31.12	33.56	27.34
DOE-SLAM	25.21	29.8	26.13

**Table 3:** Comparison of the RMSE of ATE (cm) and number of lost frames for fully dynamic object cases.

	Scenario 4		Scenario 5		Scenario 6	
	RMSE	Lost	RMSE	Lost	RMSE	Lost
ORB-SLAM2	31.30	**0**	86.98	**0**	30.60	**0**
DynaSLAM	25.76	39	16.40	31	27.34	43
DOE-SLAM	**8.61**	**0**	**16.11**	**0**	**24.73**	**0**

**Table 4:** Comparison of the RMSE of ATE (cm) in selected TUM datasets.

	ORB-SLAM2	DynaSLAM	Dynamic-SLAM	DOE-SLAM
w_static	1.74	**0.49**	-	0.58
w_xyz	1.41	1.53	1.32	**1.05**
w_rpy	6.38	**4.81**	6.03	5.71
w_halfsphere	1.79	1.77	2.14	**1.65**
s_rpy	2.40	2.02	3.45	**1.81**
s_xyz	0.99	1.17	**0.60**	0.62

**Table 5:** Comparison of the average computation time (ms) in selected TUM datasets.

	ORB-SLAM2	DynaSLAM	DOE-SLAM
w_static	17.99	23.77	29.87
w_xyz	20.89	25.28	25.11
w_rpy	20.03	24.61	28.50
w_halfsphere	19.42	27.06	26.12
s_rpy	22.08	27.06	25.48
s_xyz	20.92	24.93	26.38

**Table 6:** Comparison of the number of tracked frames in selected TUM datasets.

	Total	ORB-SLAM2	DynaSLAM	DOE-SLAM
w_static	743	646	585	**685**
w_xyz	859	638	830	**835**
w_rpy	910	727	680	**767**
w_halfsphere	1067	**1062**	1061	1061
s_rpy	820	615	455	**668**
s_xyz	1261	1194	1194	**1207**

**Table 7:** Comparison of the number of lost tracking frames.

	ORB-SLAM2	DynaSLAM	DOE-SLAM
Single moving object	**0**	22	**0**
Multiple moving objects	**0**	20	**0**

## Data Availability

Publicly available datasets were analyzed in this study. These data can be found here: TUM RGB-D dataset [https://vision.in.tum.de/data/datasets/rgbd-dataset (accessed on 29 April 2021)].
